# Validated WGS and WES protocols proved saliva-derived gDNA as an equivalent to blood-derived gDNA for clinical and population genomic analyses

**DOI:** 10.1186/s12864-024-10080-0

**Published:** 2024-02-17

**Authors:** Katerina Kvapilova, Pavol Misenko, Jan Radvanszky, Ondrej Brzon, Jaroslav Budis, Juraj Gazdarica, Ondrej Pos, Marie Korabecna, Martin Kasny, Tomas Szemes, Petr Kvapil, Jan Paces, Zbynek Kozmik

**Affiliations:** 1 Present Address: Faculty of Science, Charles University, Albertov 6, Prague, 128 00 Czech Republic; 2 Institute of Applied Biotechnologies a.s, Služeb 4, Prague, 108 00 Czech Republic; 3 Geneton s.r.o, Ilkovičova 8, Bratislava, 841 04 Slovakia; 4 Institute of Clinical and Translational Research, Biomedical Research Center of the Slovak Academy of Sciences, Dúbravská Cesta 9, Bratislava, 845 05 Slovakia; 5 Department of Molecular Biology, Faculty of Natural Sciences, Comenius University, Ilkovičova 3278/6, Karlova Ves, Bratislava, 841 04 Slovakia; 6 Comenius University Science Park, Comenius University, Ilkovičova 8, Karlova Ves, Bratislava, 841 04 Slovakia; 7 Slovak Centre for Scientific and Technical Information, Staré Mesto, Lamačská Cesta 8A, Bratislava, 811 04 Slovakia; 8 Institute of Biology and Medical Genetics, First Faculty of Medicine, Charles University and General University Hospital in Prague, Albertov 4, Prague, 128 00 Czech Republic; 9 Department of Botany and Zoology, Faculty of Science, Masaryk University, Kotlářská 2, Brno, 611 37 Czech Republic; 10 Laboratory of Genomics and Bioinformatics, Institute of Molecular Genetics of the Czech Academy of Sciences, Vídeňská 1083, Prague, 142 20 Czech Republic; 11 Laboratory of Transcriptional Regulation, Institute of Molecular Genetics of the Czech Academy of Sciences, Vídeňská 1083, Prague, 142 20 Czech Republic

**Keywords:** Genomics variant analysis, Saliva-derived gDNA, Whole genome sequencing, Whole exome sequencing, Validation guideline

## Abstract

**Background:**

Whole exome sequencing (WES) and whole genome sequencing (WGS) have become standard methods in human clinical diagnostics as well as in population genomics (POPGEN). Blood-derived genomic DNA (gDNA) is routinely used in the clinical environment. Conversely, many POPGEN studies and commercial tests benefit from easy saliva sampling. Here, we evaluated the quality of variant call sets and the level of genotype concordance of single nucleotide variants (SNVs) and small insertions and deletions (indels) for WES and WGS using paired blood- and saliva-derived gDNA isolates employing genomic reference-based validated protocols.

**Methods:**

The genomic reference standard Coriell NA12878 was repeatedly analyzed using optimized WES and WGS protocols, and data calls were compared with the truth dataset published by the Genome in a Bottle Consortium. gDNA was extracted from the paired blood and saliva samples of 10 participants and processed using the same protocols. A comparison of paired blood–saliva call sets was performed in the context of WGS and WES genomic reference-based technical validation results.

**Results:**

The quality pattern of called variants obtained from genomic-reference-based technical replicates correlates with data calls of paired blood–saliva-derived samples in all levels of tested examinations despite a higher rate of non-human contamination found in the saliva samples. The F1 score of 10 blood-to-saliva-derived comparisons ranged between 0.8030–0.9998 for SNVs and between 0.8883–0.9991 for small-indels in the case of the WGS protocol, and between 0.8643–0.999 for SNVs and between 0.7781–1.000 for small-indels in the case of the WES protocol.

**Conclusion:**

Saliva may be considered an equivalent material to blood for genetic analysis for both WGS and WES under strict protocol conditions. The accuracy of sequencing metrics and variant-detection accuracy is not affected by choosing saliva as the gDNA source instead of blood but much more significantly by the genomic context, variant types, and the sequencing technology used.

**Supplementary Information:**

The online version contains supplementary material available at 10.1186/s12864-024-10080-0.

## Background

The two key applications of next generation sequencing (NGS) technology whole exome sequencing (WES) and whole genome sequencing (WGS) principally allow the detection of most genomic variants, including single nucleotide variants (SNVs), small insertions and deletions (small-indels), copy number variants (CNVs), and tandem repeats (TRs). The WGS method showed its clear benefit in, for example, rare genetic disorders [1–3] and pediatric disorders [4] when following the updated recommendations of the EuroGenetest for WGS usage in routine diagnostics [5]. In parallel, WES has rapidly become the standard in human clinical practice [6] as an effective, economically affordable, and clinically sufficient option [7, 8]. Analysis of WGS is also used to elucidate genetic variations between populations [9], when the significance of diversity is crucial for different studies, including pharmacogenetics and pharmacogenomics [10] or polygenic risk score analysis [11, 12].

Even though saliva collection does not require skilled personnel or an appropriately equipped sampling site, most molecular diagnostic laboratories insist on the usage of genomic deoxyribonucleic acid (gDNA) extracted from whole blood (hereafter referred to only as blood). Conversely, the direct-to-consumer-genomics and even some of the population genomic projects [13] rely on the use of gDNA isolated from saliva samples obtained from self-collect sets sent by participants.

The suitability of saliva as an alternative source of gDNA for genomic analysis has been widely studied, particularly for array genotyping approaches [14, 15], including array-based methylation studies [16]. Also, WES was already referred to in the context of the usage of saliva-derived gDNA [17, 18]. However, comparisons of sequencing metrics and variant-detection accuracy among different DNA sources did not confirm saliva as a fully satisfactory source of gDNA for WGS [19]. One of the complications is the alignment of non-human reads to the human reference genome, which can result in miscalling of variant genotypes. Thus, a well-treated informatics workflow is required [20, 21]. Although the latest study [13] concluded that saliva is a suitable material for genomics population study based on WGS, all of the above-mentioned studies suffer from an imperfect approach to a comprehensive concordance analysis missing best practice recommendations—most importantly usage of internal reference standards for protocol evaluation and appropriate protocol validation [22, 23] but also compounded by other issues such as time differences in saliva and blood sampling and/or sequencing platform differences.

To test the eligibility of saliva-derived gDNA in genomic applications, we prepared an experiment to systematically compare selected sequencing parameters of saliva-derived gDNA with blood-derived gDNA, for both the WES and the WGS protocols. The accuracy of these protocols (including library preparation, sequencing, and data analysis) was evaluated in terms of their complexity and independence. For this purpose, we utilized the well-characterized National Institute of Standard and Technology human genome reference standard Coriell NA12878 (RS NA12878) and its truth dataset (TDS) and gDNA sourced from paired blood–saliva samples. Thanks to independent iterations of RS NA12878 and the existing TDS, it was possible to measure variability arising from the study protocol and compare it with the variability detected in paired blood–saliva samples. Given the complexity and specificity of CNVs and TRs analyses [24–26], this study was limited to SNVs and small-indels, and CNVs and TRs were left for future study.

## Materials and methods

### Study design

**Fig. 1:**
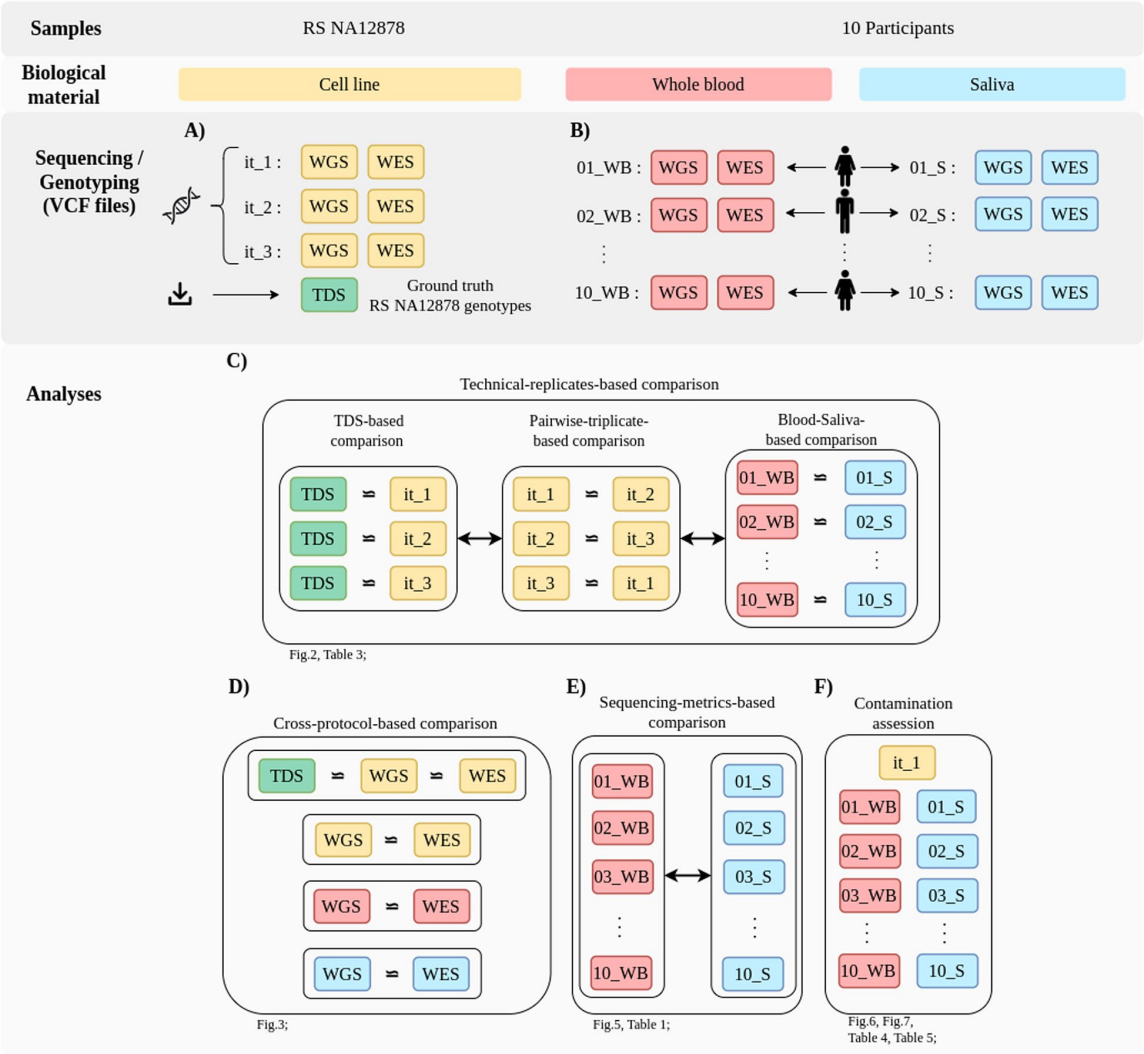
Graphical overview of the study design. **A** Biological material used in the study; **B** Sequencing analyses performed during the study; **C** Technical-replicates-based comparisons using F1 score calculations, including the pairwise-triplicate-based, TDS-based, and blood–saliva-based comparison; **D** Cross-protocol-based comparisons of concordance rates; **E** Sequencing-metrics-based comparisons; **F** Contamination rate evaluation

To perform RS NA12878-based validation, the basic technical error rate associated with the protocol (library preparation, sequencing, and bioinformatics process) was first evaluated for both WGS and WES protocols, independently. In the truth dataset (TDS) based comparison, the exact number of true positives, false positives, and false negatives was calculated against ground truth variant calls in high-confidence regions (HCR) of the RS NA12878 (genotypes provided by the Genome in a Bottle [GIAB] consortium mapped to the human GRCh38 genome reference) (v4.2.1; approx. 2,5 Tbp)) [27, 28]. Three technical replicates were sequenced of the same DNA isolate of RS NA12878 (RS NA12878_it1, RS NA12878_it2, and RS NA12878_it3), each using both WGS and WES protocols (Fig. 1A). Data evaluation was based on the determination of sequencing accuracy using F1 score calculations, either between pairwise combinations of the results of the RS NA12878 triplicates (pairwise-triplicate-based comparison) or between the available TDS for RS NA12878 and the triplicates individually (TDS-based comparison) (Fig. 1C). Concordance rates were also calculated in a cross-protocol-based manner, where variants detected using the WGS and WES for each of the replicates, as well as those in the TDS, were cross-compared (cross-protocol-based comparison) (Fig. 1D). All of these comparisons were performed using stratification to different variant types (SNVs and small-indels) as well as to different genomic contexts (genome/exome, restricted to HCR, restricted to non-difficult regions (NDR) [29, 30], or restricted to the intersection of both HCR_NDR). In addition, we also evaluated different qualitative and quantitative parameters of the sequencing experiments between the blood-derived and saliva-derived sequencing pairs (Fig. 1E). Aspects of the effect of non-human DNA contamination on sequencing were evaluated through comparisons of the ratios of reads that were mappable to the human reference genome and those that were not (Fig. 1F).

Paired blood–saliva samples were processed utilizing the same protocol in accordance with the study design (Fig. 1). The accuracy parameters of the benchmark-derived DNA sequencing data were compared with those of blood-derived gDNA and saliva-derived gDNA from sequencing data outputs of 10 individuals, including the determination of protocol accuracy using F1 score calculations of blood–saliva paired samples (blood–saliva-based comparison); these were performed individually for both WGS and WES data calls (Fig. 1B).

### Sample collection

For technical validation, the reference standard NA12878 (Coriell Institute, USA) was obtained, as a purified DNA sample, from a woman who was a participant in the International HapMap Project [31]. Three iterations of WES and WGS sequencing experiments were performed from the single aliquot of RS NA12878 extracted DNA, thus representing technical replicates of the same biological sample.

The blood–saliva paired samples were collected from each of the 10 participants (five healthy males and five healthy females with no clinical suspicion of any disease, aged 16 to 62 years at the time of sample collection) on the same day. The venous blood samples (4 mL/participant) were collected into Vacuette K2EDTA tubes (Becton Dickinson, USA), tempered for 30 min to room temperature, and then stored at 4 °C. To minimize the ratio of non-human DNA contamination in the saliva samples, the saliva collection was strictly controlled. Participants washed their teeth by toothbrush without toothpaste for approx. 1 min., rinsed their mouths with water, and avoided eating or drinking for 30 min before collecting the sample. A total of 2 × 1 ml of saliva was collected from each participant into 4 mL of PBS pH 7.2 (HiMedia, Germany) including PSA (penicillin 100 IU/ml, streptomycin 100 µg/ml, amphotericin B 2.5 µg/ml, sodium deoxycholate 2.5 µg/ml; Serana, Germany) and stored at 4 °C.

### Genomic DNA extraction

All gDNA extractions were performed within 24 h after sample collection. The gDNA from blood and saliva samples were isolated using the isolation kit QIAamp® DNA Blood Mini Kit (QIAGEN, Germany) with a sample origin-dependent protocol. Blood-derived gDNA isolates were obtained from 1 mL of collected blood samples according to the manufacturer's protocol "DNA Purification from Blood or Body Fluids," whereas saliva-derived gDNA isolates were extracted from 2 mL of saliva-media mixture according to the modified QIAGEN Supplementary Protocol “Isolation of genomic DNA from saliva and mouthwash using the QIAamp® DNA Blood Mini Kit, Spin procedure protocol.” The purity of gDNA was evaluated based on the A260/280 absorbance ratio acquired spectrophotometrically on NanoPhotometer P300 (Implen, Germany) from 1 µl of gDNA isolates. The concentrations of gDNA were measured in duplicates (2 × 1 µl) using the Qubit 1 × dsDNA High Sensitivity (HS) Kit (Thermo Fisher Scientific, USA) (Additional File 1, Table 1). The integrity of gDNA was evaluated using the 0.8% agarose gel electrophoresis (GelRed 1:10 000, 100 V, 40 min), (Additional File 2, Fig. 1).

### NGS library preparation and quality control

Whole genome libraries were prepared using the TruSeq DNA PCR-Free kit (Illumina, USA) with standard input of 1 µg of gDNA, employing mechanic fragmentation on the Covaris M220 (Covaris, USA) as described by the manufacturer's guide. Whole exome libraries were prepared from 100 ng of gDNA using the Illumina DNA Prep with Enrichment (Illumina, USA) library preparation kit in combination with panels for hybridization-capture: the Alliance VCGS Exome panel and Mitochondrial DNA panels (both Twist Bioscience, USA). The concentration of WES/WGS libraries was measured in duplicates (2 × 1 µl) using the Qubit 1 × dsDNA HS Kit (Thermo Fisher Scientific, USA). The quality of NGS libraries was analyzed using the automated electrophoresis on the 2100 Bioanalyzer System with DNA HS chip (Agilent, USA) (Additional File 1, Table 1).

### Sequencing

The quantity of non-human content of prepared blood, saliva, and RS NA12878_it3 WGS libraries were assessed by low-pass pre-sequencing on an iSeq 100 sequencer (Illumina, USA). For this, one pool of WGS libraries (20 paired samples + NA12878_it3) was diluted and denatured according to the iSeq 100 sequencing guide and pre-sequenced on iSeq using the i1 Reagent v2 (300 cycles) sequencing kit (Illumina, USA) in pair-end mode (2 × 150 bp). Based on the pre-sequencing results, the pooling of whole genome libraries was adjusted to deliver average output ≥ 800 million pair-end reads of human reads = average coverage ≥ 30 × for each sample (Additional File 1, Table 2). In line with this, four whole genome library pools (20 paired samples + NA12878_it3) were prepared based on the results of pre-sequencing on the iSeq 100. The NGS libraries pools were diluted and denatured according to the NovaSeq 6000 Denature and Dilute Libraries Guide (Illumina, USA). The individual pools were sequenced using S4 Reagent Kit v1.5 (300 cycles) (Illumina, USA) with the XP4 workflow in one pool/one line mode. A sequencing run with a 2 × 151 cycles configuration was performed on the NovaSeq 6000 (Illumina, USA). The WGS libraries of RS NA12878_it1 and RS NA12878_it2 were sequenced in two separated S4 (300 cycles) runs. For the hybridization-based WES enrichment, the libraries originating from saliva-derived gDNA were adjusted in DNA input quantity for pooling in plex according to the tested portion of human reads in these iSeq runs (Additional File 1, Table 1). Sequencing of WES libraries resulted in targeted ≥ 60,000 pair-end human reads =  ≥ 100 × average coverage per sample (Additional File 1, Table 2).

### Bioinformatic processing of the sequencing data

#### Primary analysis

Files in BCL format were converted into demultiplexed FASTQ-format files using bcl2fastq v2.20.0.422 with default settings and one barcode mismatch allowed. Quality control of the FASTQ files was done in FastQC [32]. The fastp tool [33] was used for quality trimming and removal of the adapters and sequencing artifacts from both the WES and WGS datasets.

### Secondary analysis

The WES data were analyzed with the DRAGEN Bio-IT Platform DNA ENRICHMENT pipeline v3.10. (Illumina, USA), while the WGS data were analyzed with the DRAGEN Bio-IT Platform DNA GERMLINE pipeline v3.10 (Illumina, USA), both with default settings for mapping and variant calling (SNVs and small-indels up to 1 000 bp) [34]. Sequencing reads were aligned to the Illumina DRAGEN Graph human GRCh38 genome reference (alt-masked-v2). All reads with mapping quality > 1 were used for the variant calling. The “hard” filter was used for variants quality filtration with the following thresholds: QUAL > 10.4 and AF > 5%. The WES data analysis was performed in target regions defined by the Twist Alliance VCGS Exome panel (Twist Bioscience, USA) merged with the Twist Mitochondrial Panel (Twist Bioscience, USA) (browser extensible data [BED] files available on:https://www.twistbioscience.com/resources/data-files/twist-alliance-vcgs-exome-401mb-bed-files and https://www.twistbioscience.com/resources/data-files/twist-mitochondrial-dna-panel-bed-files). For mapping and variants calling QC, the DRAGEN metrics were used together with the output of the vcfstats tool [35].

### Contamination analysis

The FastqScreen tool [32] was used to analyze the proportion of microbial reads from all unmapped reads by mapping them against an oral human microbiome database [36]. This analysis includes automatic subsampling of the reads.

### Protocol validation

To determine the accuracy of the complete WGS and WES protocols, the F1 scores calculated from precision (truth positive predictions relative to total predicted positives) and recall (truth positive predictions relative to total actual positives) of observed variants were used. The F1 score ranges between 0–1, with a value of 1 indicating complete agreement between calls from two compared variant/genotype datasets [37, 38]. For the validation of the WGS and WES protocols in the study, whole genome and whole exome libraries from the RS NA12878 in triplicates (RS NA12878_it1, RS NA12878_it2, RS NA12878_it3) were prepared. Sequencing accuracy, in terms of F1 score calculations, was performed using the comparisons of variant call format (VCF) files: 1) of pairwise combinations of the results of the RS NA12878 triplicates (pairwise-triplicate-based comparison); 2) between the available TDS for RS NA12878 and the triplicates individually (TDS-based comparison). Similarly, data generated for blood–saliva paired samples were also compared (blood–saliva-based comparison). In the TDS-based comparison, the exact number of true positives, false positives, and false negatives was calculated against ground truth variant calls in HCR of the RS NA12878 (genotypes provided by the Genome in a Bottle [GIAB] consortium mapped to the human GRCh38 genome reference) (v4.2.1; approx. 2,5 Tbp)) [27, 28]. The comparison of the VCF files generated from WGS and WES data calls was done with the hap.py tool (version 0.3.14) [39]. The GRCh38 was used as a reference in the hap.py analysis. Furthermore, switches “–preprocess-truth” and “–usefiltered-truth” were used with each hap.py analysis. The F1 score median value was calculated from three experiments separately for the whole genome, high confidence regions (HCR), non-difficult regions (NDR) (v3.0, approx. 2,3 Tbp) [29, 30], and for the HCR and NDR intersection (HCR_NDR) of GRCh38. To characterize problematic genomic regions, we decided to evaluate F1 scores in the intersection of the difficult-to-sequence region (GRCh38 outside of NDR) and of the GIAB low-confidence region (GIAB outside HCR), marking it in the following text as HARD.

Concordance rates were also calculated for variants detected using the WGS and WES protocols for each of the replicates individually, and also for the WGS and WES limited to TDS (cross-protocol-based comparison). This cross-protocol-based comparison was also performed for the blood–saliva pairs. To define the concordance of WGS and WES data calls, we generated an intersect BED file "file4truth" (available upon request) by intersecting the target regions BED file of the Twist Alliance VCGS Exome panel, HCR of RS NA12878 and NDR of GRCh38. BEDTools (v2.30.0) was used for intersecting, merging, and sorting the final BED file. The genotype analysis of matching variants between multiple methods was executed using a series of sequential steps. First, each variant file was preprocessed by the pre.py tool using the same reference genome as with the previously described hap.py comparison. The resulting preprocessed file was then restricted to the regions specified by the "file4truth," utilizing the “bcftools view” with the filter “–apply-filters ‘PASS’.” Subsequently, all desired variant files were merged together using the bcftools merge, applying the following parameters: “–apply-filters ‘PASS’,” “–force-samples,” and “–merge both.” This facilitated the creation of a single unified variant file. Subsequently, to determine the number of matching variants in each subset, a proprietary Python script was used to count variants in the final unified variant file. Finally, upsetplots were created using the Python package upsetplot (v0.8.0).

### Data analyses and statistical methods

Pairwise concordances (measured by F1 score) between various experimental conditions were compared using mixed analysis of variance (ANOVA) implemented in the pingouin Python package. Post-hoc tests were used to calculate the statistical significance of individual conditions. Obtained *p*-values were further corrected for multiple hypotheses by the step-down method using Bonferroni adjustments (method pingouin.pairwise_tests, parameter “padjust = 'holm'”).

The comparison of sequencing metrics between groups of blood and saliva samples was performed using the non-parametric Mann–Whitney test, which was implemented using the SciPy Python package.

Data analysis and visualization presented in this publication were performed in Python 3.8.13 using the following packages: scipy 1.7.3 [40], matplotlib 3.5.2 [41], numpy 1.21.5 [42], pandas 1.4.4 [43], pingouin 0.5.2 [44], seaborn 0.11.2 [45]. Circular genomic graphs were generated by Circos v 0.69–8 [46] running Perl 5.032001.

## Results

### Blood–saliva concordance rate estimations in the context of the technical validation of WGS and WES protocols

#### Technical-replicates-based comparisons of accuracy with or without the use of TDS

**Fig. 2:**
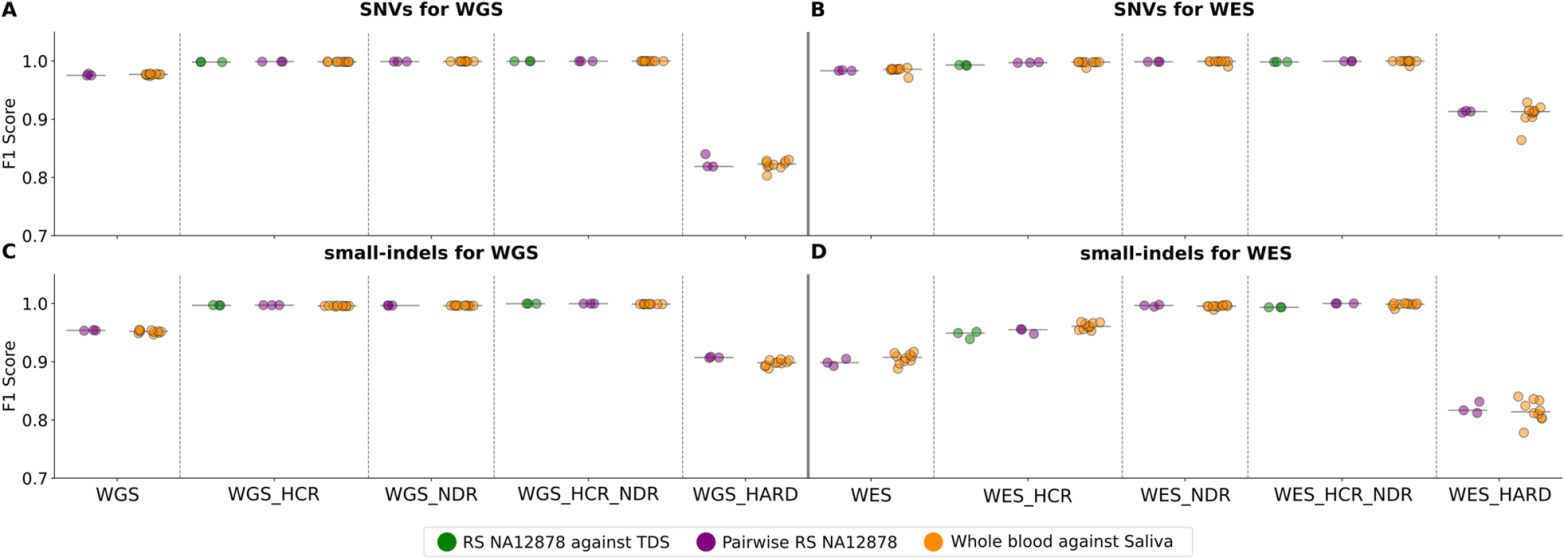
Results of comparisons shown as F1 scores for WGS and WES analyses individually. Results are arranged into four sections according to the used protocol and variant type. Each group contains plots for the five different genomic regions evaluated, i.e., whole reference genome or exome (WGS or WES), high-confidence regions (WGS_HCR or WES_HCR), non-difficult regions (WGS_NDR or WES_NDR), HCR and NDR intersection (WGS_HCR_NDR or WES_HCR_NDR), and problematic genomic regions (WGS_HARD or WES_HARD). Horizontal lines in each group represent the median value for a particular group. Data points represent individual samples inside each particular group. Dispersion of data points along the x axis inside each particular plot does not represent a range of values on this axis, rather a visual aid to allow better resolution of the data points

In this part of the study, the technical error rate of the sequencing process itself was determined (technical validation) for WGS and WES protocols independently, using two complementary approaches: pairwise-triplicate-based and TDS-based. The same analyses were also performed for the blood–saliva pairs, and the resulting F1 scores were compared with those obtained in the technical validation results. The results are summarized in Fig. 2 and Additional File 1, Table 3. Each concordance rate was determined individually for SNVs and for small-indels. In addition, comparisons were performed on different parts of the genome: on the whole GRCh38 reference genome (labeled as WGS) or exome for the WES protocol (labeled as WES), or; with restrictions to the HCR region of the RS NA12878 (WGS_HCR or WES_HCR); to the NDR (WGS_NDR or WES_NDR); with restrictions to the intersection of HCR and NDR (WGS_HCR_NDR or WES_HCR_NDR); and also with restrictions to problematic genomic regions (WGS_HARD or WES_HARD). All previous comparisons were calculated for autosomes only. HCR_WGS of the RS NA12878 represented 87.38% of the genome, while HCR_WES was 90.28% of the exome. NDR_WGS accounted for 75.77% of the genome, whereas NDR_WES accounted for 71.92% of the exome. Additionally, HCR_NDR_WGS accounted for 73.89% of the genome, whereas NDR_HCR_WES accounted for 67.64% of the exome. WGS_HARD and WES_HARD accounted for 9.99% and 4.18% of the genome or exome, respectively. All were calculated for autosomes only.

For the WGS protocol, when considering all of the tested possibilities, the determined F1 scores ranged between 0.8030–0.9998. Similarly, when considering all tested possibilities for the WES protocol, F1 scores ranged between 0.7781–1.000. It should be noted that the calculated scores for both the WES and WGS protocols exhibited a strong dependency on specific variable combinations. Importantly, F1 scores determined for the pairwise-triplicate-based and for the TDS-based validation approaches, as well as for the blood–saliva-based comparisons, tended to have very similar median values and comparable distributions (Fig. 2; Additional File 1, Table 3). For example, for WGS, when comparing F1 scores from blood–saliva pairs and those determined from the pairwise-triplicate-based comparisons, the differences reached no statistical significance for SNVs (*p* = 0.609, pointing to virtually no difference between the groups). Also, for small-indels, the differences between the groups achieved only a very weak statistical difference (*p* = 0.014) (Fig. 2A, C; Additional File 1, Table 3).

When evaluating the effect of variant types and genomic regions on WGS data calls, we found that for pairwise-triplicate-based, and also for the TDS-based results, SNVs resulted in higher accuracy than small-indels in all analyzed genomic regions (*p* = 0.0025 when comparing SNVs and small-indels among pairwise-triplicate-based results throughout each genomic region) (Fig. 2A, C). The lowest F1 score was found in SNVs when the genomic context was restricted to the WGS_HARD (min. F1 = 0.8030). In contrast, the highest values achieved were determined when restricting the region of interest to WGS_HCR_NDR (max. F1 = 0.9998). Here, the differences between the F1 scores of SNVs and small-indels were the smallest. The main increase in accuracy was visible when restricting the genomic region of interest from the WGS either to WGS_HCR or to WGS_NDR, or even more markedly to the WGS_HCR_NDR.

With regard to the accuracy of the WES protocol in general, patterns similar to the WGS protocol were obtained (Fig. 2B, D). This holds for the comparability of pairwise-triplicate-based technical validation results to those obtained by WES on blood–saliva pairs, as well as for the effect of variant types and genomic context. Small-indels led to an expected decrease in accuracy when compared with SNVs (*p* = 0.00003 considering all genomic regions). Limiting the genomic context from WES to WES_HCR led to higher median F1 scores, but for small-indels these still did not achieve the median F1 scores of SNVs in these regions. For small-indels, limitation to WES_NDR was necessary to achieve median F1 scores comparable with SNVs. The lowest F1 score was achieved for small-indels in the WES_HARD region (F1 = 0.7781), while the highest (F1 = 1.000) in the WES_HCR_NDR. Importantly, similarly to the WGS protocol, the WES protocol led to highly comparable F1 scores obtained from the pairwise-triplicate-based technical validation to those obtained from the blood–saliva comparisons (*p* = 0.810 for SNVs and *p* = 0.722 for small-indels and both considering results throughout all genomic regions). However, it should be noted that the distribution of F1 scores was much more heterogeneous in the WES protocol when compared with the WGS results, while this was more pronounced in small-indels as well as in the whole WES and WES_HCR regions.

#### Cross-protocol-based concordance rate estimations with or without TDS

**Fig. 3:**
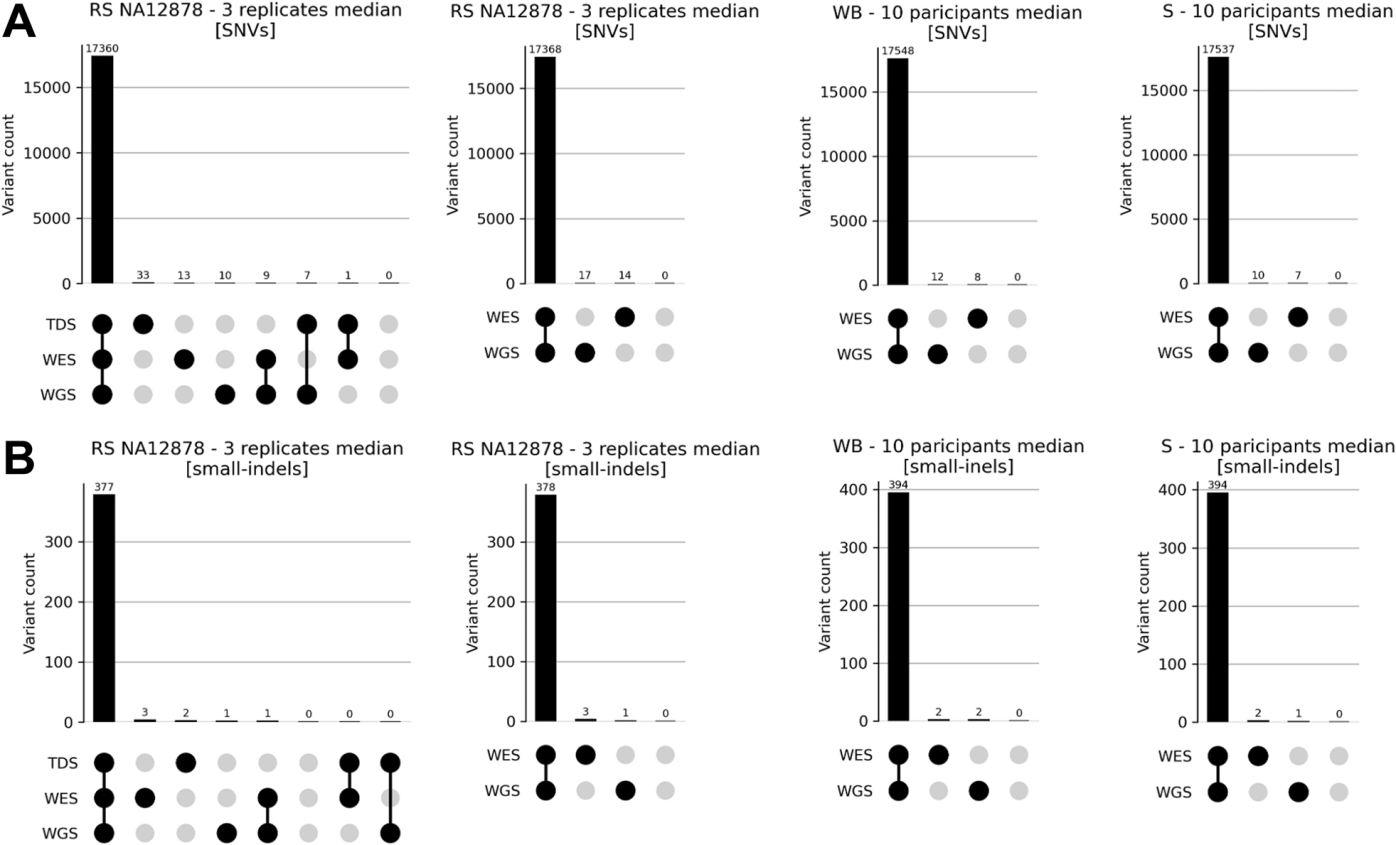
Analysis of genotype concordance of the three RS NA12878 iterations, as well as of the blood and saliva samples. **A** results for SNVs; **B** results for small-indels; WB—whole blood, S—saliva. Concordance rate calculations were performed using an agreement analysis between RS NA12878, WB, and S, all limited to autosomes and HCR. To ensure a consistent comparison of results in WES and WGS regions between WB and S analyses, RS NA12878 was subjected to both TDS-inclusive and TDS-exclusive analyses

In the second comparison approach, genotype concordance rates were determined between the defined genotypes in the TDS and the WGS and WES results of the three iterations of the RS NA12878, all restricted to autosomes (to eliminate sex chromosome-related genetic male–female differences) and to the HCR. For this, we used the “file4truth” BED file that defines their intersection. In the “file4truth,” the extent of intersecting genomic regions was 27,031,362 bp. In the three RS NA12878 iterations, the median of the variant numbers concordantly genotyped by the WGS and WES protocols, which are present also in the TDS, were 17,360 for SNVs and 377 for small-indels, with a concordance rate of 99.58% and 98.43%, respectively. Comparable numbers of SNVs (17,368) and small-indels (378) were identified when considering the WGS and WES protocol results alone (not considering the TDS variants), with a concordance rate of 99.82% and 98.95%, respectively. Compared to this, 17,548 SNVs and 394 small-indels were concordantly genotyped using WGS and WES in the blood samples (median values; concordance rate of 99.89% and 98.99%, respectively), while 17,537 SNVs and 394 small-indels were concordantly genotyped using WGS and WES protocols in the saliva samples (median values; concordance rate 99.90% and 99.24%, respectively). The results of these comparisons are summarized in Fig. 3, Additional File 1, Table 4.

#### Distribution patterns of sequencing accuracy on the genomic background

**Fig. 4:**
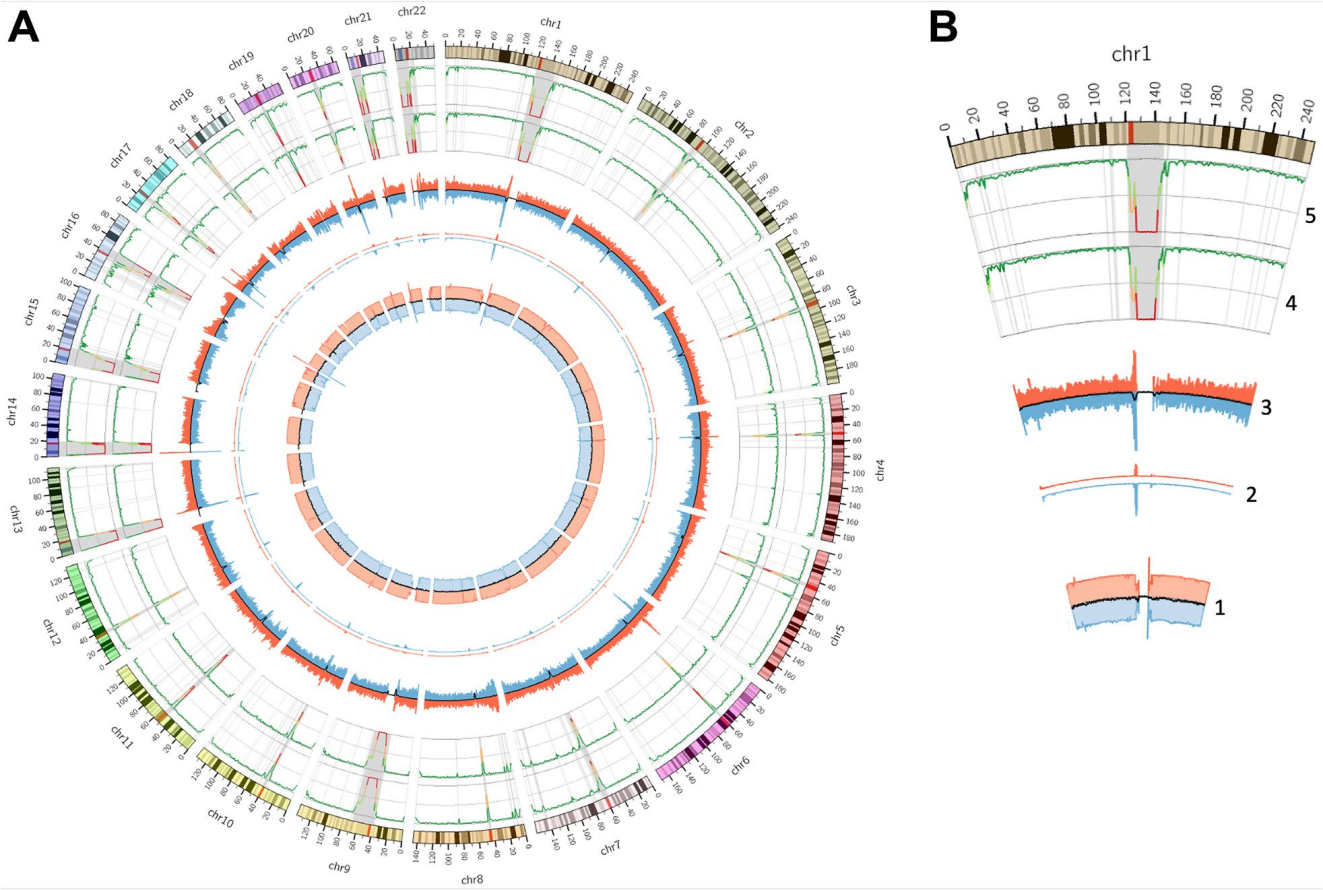
Coverage, variant, and F1 score distributions throughout the human reference genome. Characteristics are clustered by 1,000,000 bases. Gray regions outline problematic genomic regions (HARD). The axis of each red track corresponds to low values to high values from the inside to the outside. The axis of each blue track corresponds to low values to high values from the outside to the inside. Track numbering from the inner circle: Track 1: Average genome coverage of saliva (blue) and blood (red) samples. Reads filtered for quality > 10; Track 2: Average count of variants unique for saliva (blue) samples and variants unique for blood (red) samples; Track 3: Average count of all variants in saliva (blue) samples and all variants in blood (red) samples; Track 4: Median F1 score of comparisons between RS NA12878 repetitions; Track 5: Median F1 score of saliva versus blood comparisons. **A** Whole genome view (excluding gonosomes); **B** Zoom on chromosome 1. For better visual resolution, please find the zoomable online version of this Figure (Additional File 2, Fig. 2)

To characterize the potential discrepancies between the blood and saliva samples, different variant-related parameters from the data against chromosomal locations and sequencing coverage were plotted (see Fig. 4). Patterns of fluctuations in mean values of F1 scores in saliva vs. blood were very similar to the patterns found in RS NA12878 triplicates. In addition, both patterns reflected not only the distribution of difficult-to-sequence regions in the genome but also of the average counts of all identified variants. In addition, the unique variants identified in both saliva and blood also tended to cluster in the same regions. These clustering patterns are very similar to the strongest prevalence of difficult-to-sequence regions, where the F1 scores also tend to drop most significantly, and where the biggest fluctuation in coverage is visible.

### Sequencing-metrics-based blood–saliva comparison

**Fig. 5:**
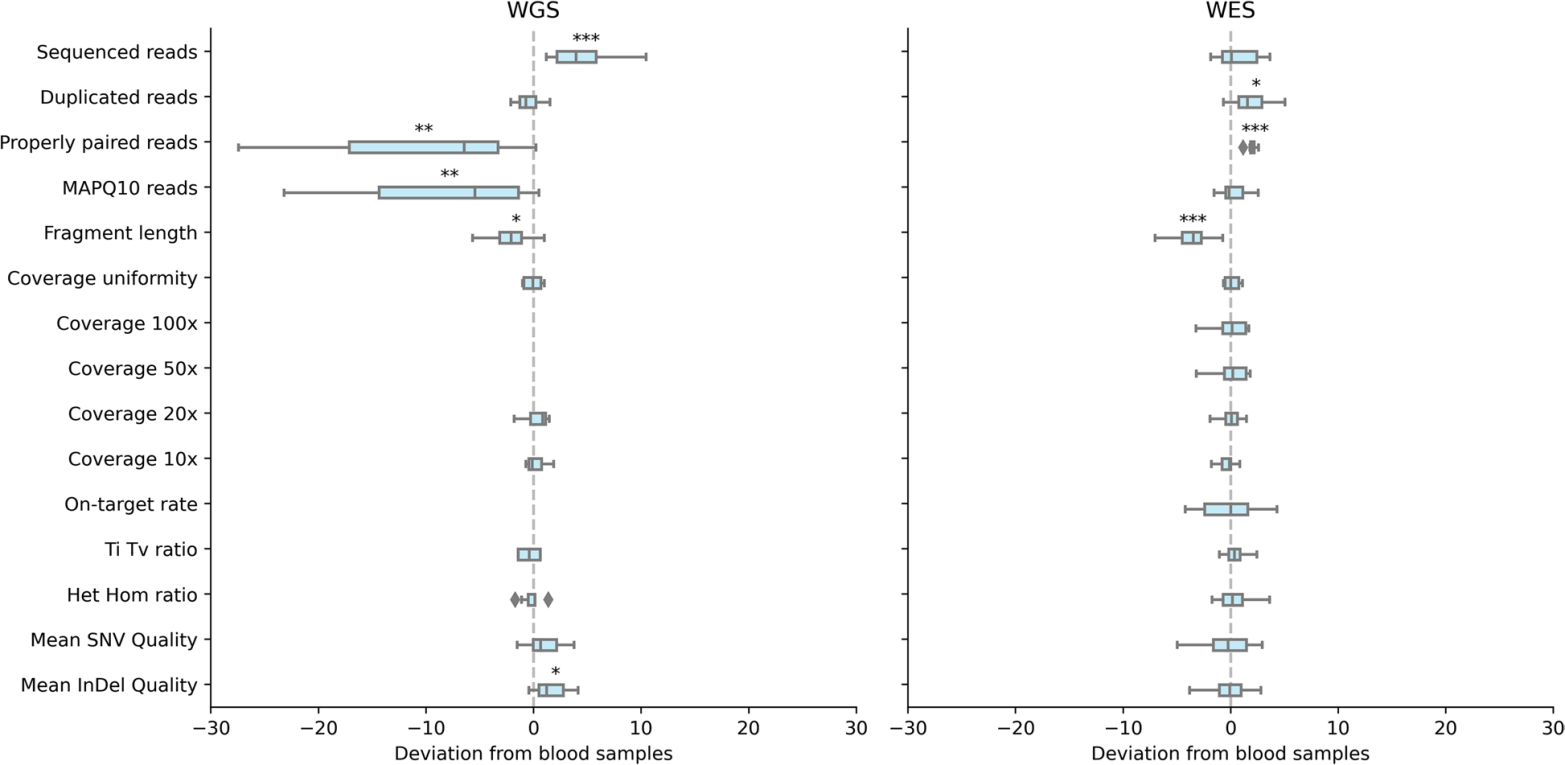
The quality control metrics of sequencing runs. Results of saliva-derived DNA samples (box plots) are compared with those of blood-derived samples (the relative mean is represented as a vertical dashed line at x = 0). Deviations of saliva samples, shown on the x-axis, are determined using standard Z scores, which represent the number of standard deviations away from the mean of blood-derived samples. The stars displayed above the box plots indicate the level of significance of the differences between the two sample types, with (*) denoting *p* < 0.05, (**) *p* < 0.01, and (***) *p* < 0.001. The largest discrepancy was observed in the proportion of mapped reads, as depicted in the lower plot. Comparisons without statistical significance have no stars

The third blood–saliva comparison was based on independent comparisons of sequencing metrics among blood–saliva pairs for the WGS and WES protocols (Fig. 5; Additional File 1, Table 2). The majority of metrics, such as duplicated reads, coverage uniformity, coverage depths (compared at different depths from 10x–100x), on-target rate, transition-transversion ratio, and heterozygous-homozygous ratio, were concordant between blood- and saliva-derived gDNA sequencing results, for both the WGS and the WES protocols. The numbers of sequenced reads were found to be higher in saliva when compared with blood, but this was statistically significant only for WGS (and, moreover, expected based on the study design). Variant calling quality for both SNVs and small-indels was found to be higher in saliva, especially in WGS, but this was statistically significant only for small-indels. Mapped reads, on the other hand, were found to be lower in saliva, with statistical significance in both WGS and WES data calls; however, this was more prominent for WGS. Duplicated reads were also higher in saliva samples; however, this was statistically significant only for WES. There were fewer MAPQ10 reads in saliva samples but with statistical significance only in WGS data. Fragment lengths were lower in saliva, with statistical significance both in WGS and WES.

### Contamination rate

**Fig. 6:**
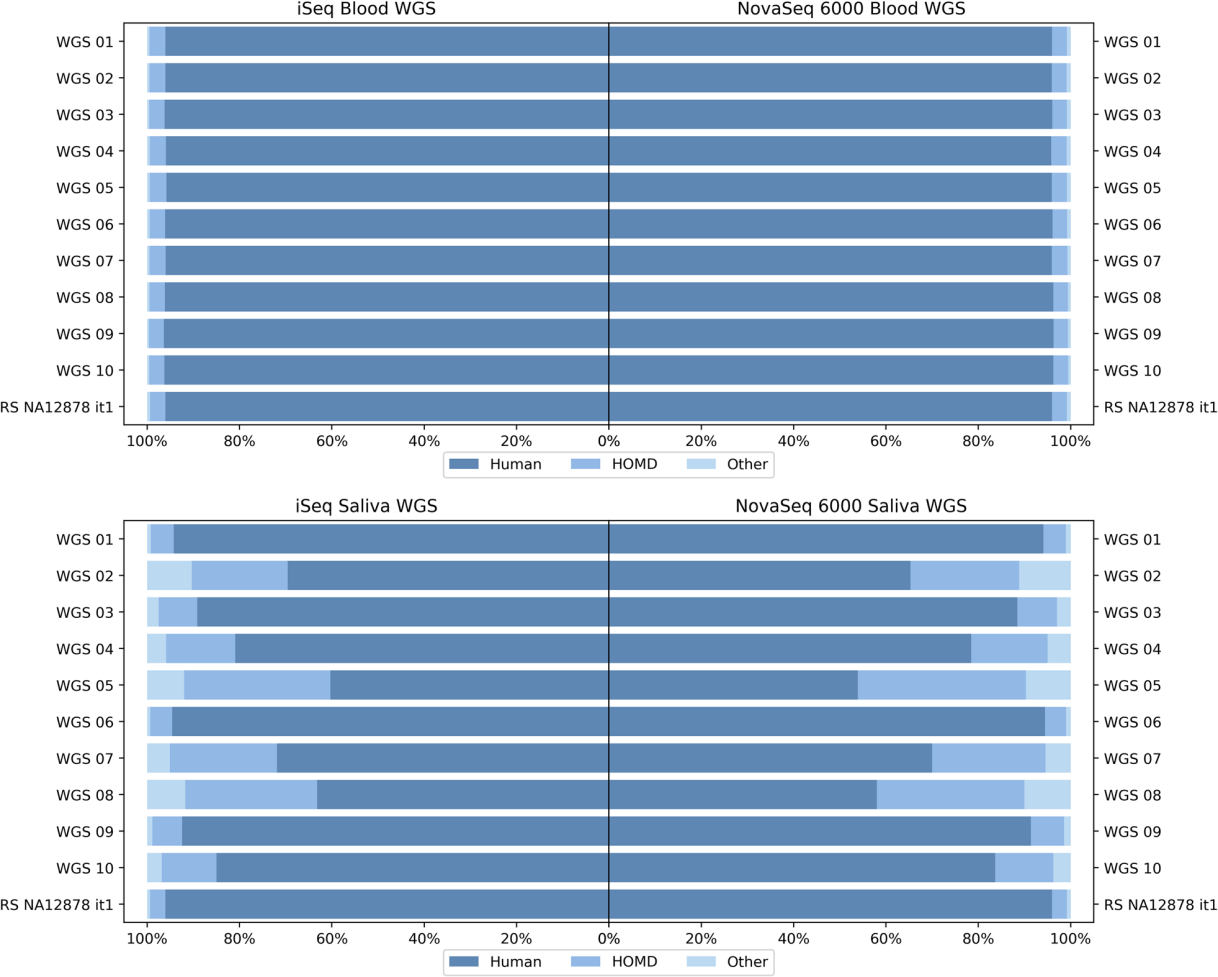
Evaluation of WGS read mapping results with a focus on the mappability to the GRCh38 human reference genome and on the HOMD, both for the iSeq pre-sequencing and for the final NovaSeq6000 sequencing results. It should be noted that the NovaSeq 6000 reads were subsampled during the processing of the data for this comparison. **A** Blood-derived gDNA WGS on iSeq vs. NovaSeq 6000 visualized as proportions of reads; **B** Saliva-derived gDNA WGS on iSeq vs. NovaSeq 6000 visualized as proportions of reads; HOMD = Human Oral Microbiome Database

Contamination detection was performed to assess the ratio of human and non-human sequencing reads in the saliva-derived gDNA samples. This was performed twice during the study: first, in the time of the WGS library preparation, when the contamination ratio (iSeq experiment) was used to calculate pooling ratios of individual samples to obtain a comparable number of human-mappable reads between samples; second, it was performed from the resulting NovaSeq 6000 experiment to check the final ratio. Notably, both the iSeq monitoring experiment and the final NovaSeq 6000 experiment revealed highly similar relative contamination rates, and this was typical for both the blood and the saliva samples (Fig. 6).

**Fig. 7:**
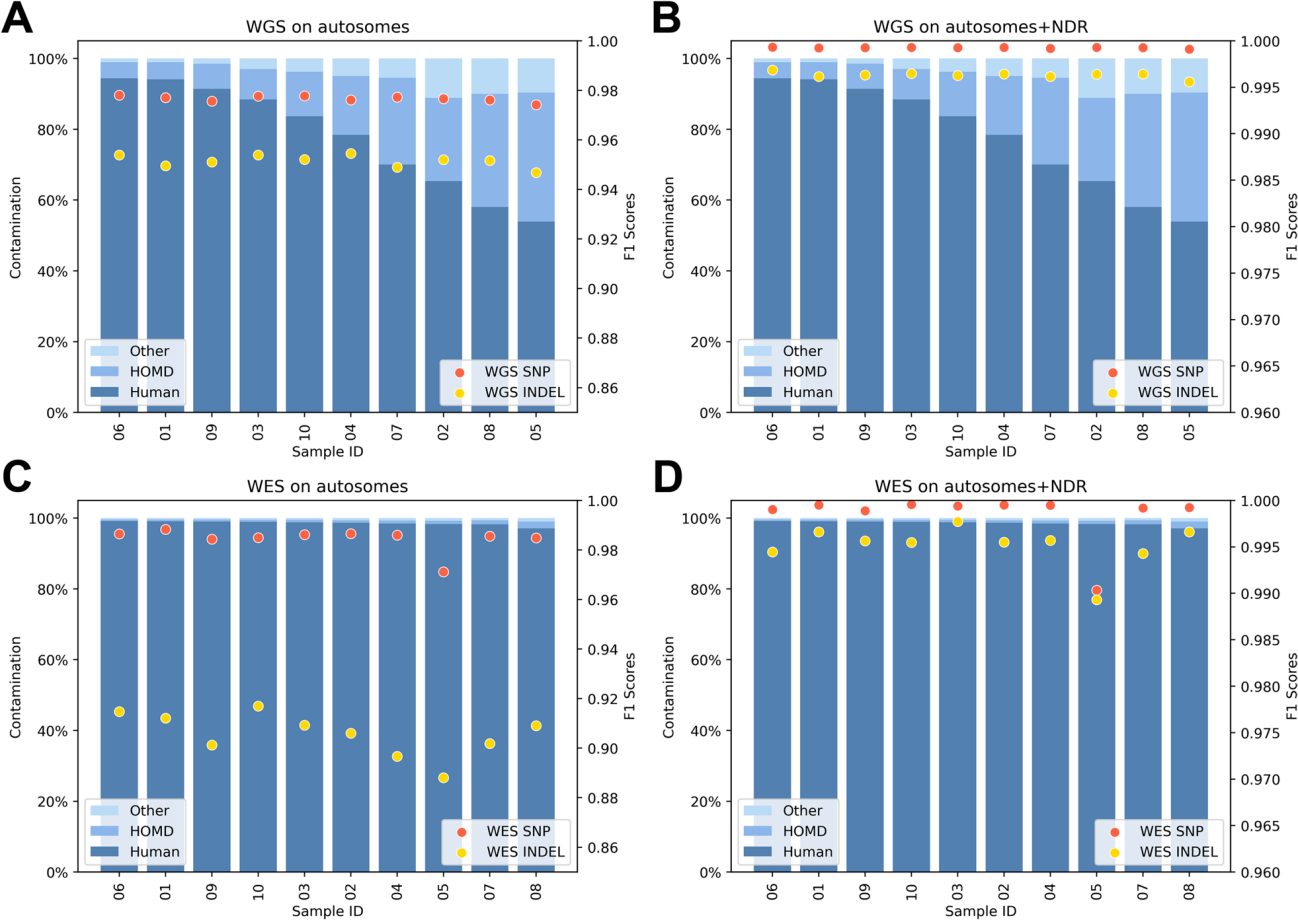
Comparison of read-mapping results of the saliva-derived gDNA samples (bar plots) and the F1 scores of these samples (red dots for SNVs and yellow dots for small-indels). Saliva-derived gDNA samples (bar plots) with percentual values on the left y-axis; F1 scores (green lines for SNVs and red lines for small-indels) with values on the right y-axis. **A** WGS results; **B** WGS results limited to NDR; **C** WES results; **D** WES results limited to NDR. HOMD = Human Oral Microbiome Database

As expected, higher non-human contamination was detected in the saliva samples. Also, the in-between sample heterogeneity in saliva samples analyzed by the WGS protocol was observed to be high, despite the strict control of the collection process. An analysis of the saliva samples revealed that 8%–45% of WGS reads did not map to the human GRCh38 genome reference, whereas in the case of RS NA12878_it1–3 and blood-derived samples this was 4%–5%. Although inter-sample variability remained relatively high when considering both total read numbers and relative contamination rate, pooling of libraries based on their respective contamination rate estimated based on iSeq pre-sequencing results achieved the minimum required numbers of human-mappable reads in all saliva samples (in accordance with the study design), comparable with those obtained from the blood samples and also to the benchmark standards. The WES library preparation protocol strongly reduced the number of non-human-mappable reads in both the blood and saliva samples; however, in the saliva samples, the inter-sample heterogeneity was still higher when compared with blood samples (Additional File 1, Table 5; Additional File 2, Fig. 3). Importantly, plotting F1 scores against relative contamination rates in individual samples did not reveal trends that would suggest any effect of contamination rates on sequencing accuracy (Fig. 7; Additional File 1, Table 6).

## Discussion

Since genetic and genomic testing using massively parallel sequencing is becoming commonplace, not only in research and laboratory diagnostics but also in direct-to-consumer-based lifestyle genomics, with a high impact on everyday life, it is vital to ensure the quality and comparability of generated genomic data. Developed professional guidelines and protocols underline the ever-increasing importance of thorough validation of these complex processes, including both the wet lab and the in silico parts [23, 29, 47]. How to exactly perform these validation steps is a subject of extensive debate, with several alternative suggested approaches. Although there is no single procedure that is without drawbacks or inefficiencies, creating benchmarking datasets for technical validation processes is one of the emerging strategies [27, 48]. Whether the biological source of the genomic material to be analyzed has any consequences on results is also widely discussed, with several contradicting results published [49].

The main aim of this study was to thoroughly evaluate the reliability of genomic testing accuracy for germline sequence variant detection performed on saliva-derived gDNA, with a special focus on comparing the results with those obtained from blood-derived gDNA from the same individuals. Both types of samples have different content of cellular material. Moreover, saliva samples contain various cells such as epithelial cells, leukocytes, and microorganisms in different proportions from sample to sample [50]. Leukocyte content may make saliva samples the best alternative material to blood compared with other mouth samples. To determine this, it was necessary to determine first the technical reliability of the WGS and WES protocols, including both the used wet lab (from library preparation) and bioinformatic pipelines (up to variant calling), as is generally recommended [47]. Therefore, a commercial benchmark DNA standard, RS NA12878, was sequenced in triplicate, using both the WGS and WES protocols. The generated data calls were processed using a unified bioinformatic pipeline up to the secondary analysis, i.e., up to the generation of VCF files for SNVs and small-indels. In parallel, blood–saliva samples from ten individuals were sequenced, again using both the WGS and WES protocols and with the same bioinformatic pipeline. It is important to note that the biological source of the benchmark DNA sample is different from those used in the blood–saliva comparisons, as it is isolated from cultured cell lines. Such benchmark samples are generally considered a best-practice choice in validation processes for genomic sequencing [29]. Consistently with this, performance and quality results were determined on the triplicates of the cell-line-based benchmark standard RS NA12878 so that they were valid for the blood-derived DNA samples, which is a commonly used practice. At the same time, the results of the blood-derived DNA samples were used to evaluate the performance and quality metrics of the saliva-derived DNA samples.

When discussing technical validation approaches and sequencing accuracy, comparisons to TDS are considered the gold standard, even if there are still some technical limitations [29]. In addition to a TDS-based comparison, additional validation forms were tested, such as a comparison of technical replicates sequenced using the same protocol (WGS or WES results evaluated separately) and also a comparison of results obtained using different sequencing protocols (a WGS and WES results cross-comparison); while both these latter cases involved no a priori knowledge from a TDS. There were several reasons for this, including the following: 1) the TDS-based approach is limited to the HCR, even after efforts to continuously extend the HCR for the available RS NA12878 [51]; 2) the same variants may be represented in VCF files differently, leading to false differences between the compared variant sets; 3) there were no saliva samples or saliva-derived gDNA samples available as the benchmark standards; and 4) real differences between the TDS and the RS NA12878 may also exist, since the RS NA12878 batch was derived from cell lines which went through several passages when compared with those that were used for the TDS determination, presumably accumulating sequence variants. The latter may bring biological-sample-based systematic errors, theoretically leading to underestimated accuracy. On the other hand, both technical-replicate-based and cross-protocol-based methods may overestimate accuracy because of the possible systematic effect of process-, protocol-, or platform-specific errors (technical-systematic-errors).

In fact, the pairwise-triplicate-based results seem to have higher F1 scores than the TDS-based ones, both in WGS and WES, but in WES this was found to be more pronounced (Fig. 2 and Additional File 1, Table 3). It is, however, hard to say, what are the roots of this difference. The biological-sample-based systematic errors mentioned above, potentially creating underestimated accuracy, would presumably result in discordant positions which are either present in the TDS but are missing in both the WES and WGS results, or are missing in the TDS and are present in the WES and WGS results (Fig. 3, Additional File 1, Table 4). These could, on the other hand, also represent technical-systematic-errors which affect in the same way both the WGS and WES protocol, creating real false positives/negatives against the TDS. In line with this, when examining genotype concordance rates between the TDS and the individual RS NA12878 iterations, slightly higher numbers of variants being found only in the TDS, or only in the three iterations (in all of them but missing in the TDS), were visible, when compared to other concordance combinations. This trend was similar both for SNVs and small-indels, and both in the WGS and in the WES data (Additional File 2, Fig. 5). Identifying the real roots of these discrepancies would require, however, specific validation analyses. Other combinations from the cross-protocol-based comparison, explicitly suggesting real sequencing errors (such as variants found only in the WGS or WES results, or in combinations of TDS with either WGS or WES), being less prevalent than the above mentioned types of discrepancies.

Finally, although either the TDS-based, or the technical-replicate-based, or the cross-protocol-based validation approaches have their pros and cons, it should be stressed that the aim of the study was not to ultimately determine the sequencing accuracy per se*.* Rather, the intention was to determine, using the same validated experimental protocol, the usability of saliva-derived gDNAs: in particular, whether they are comparable in accuracy for genomic and genetic analyses to those that are blood-derived. Moreover, the pairwise-triplicate-based approach allows the determination of the protocol accuracy not only for the HCR but also outside this region, which is often unobtainable from TDS-based validation data alone. In addition, the analyses on the RS NA12878 suggest that the TDS-based results are comparable with those obtained using the pairwise-triplicate-based and also using the cross-protocol-based results. In line with these, supported by these findings, we concluded that the validation approaches that do not use TDS may be considered representative and can be used to evaluate the blood–saliva comparisons.

F1 scores determined from the blood–saliva-based comparisons tended to have very similar median values and distributions to those determined from the RS NA12878 pairwise-triplicate-based comparisons. Also, the numbers of concordant and discordant variants in the cross-protocol-based comparison were found to be comparable between the technical replicates and the blood and saliva samples. In addition, variant calling quality was found to be even slightly higher in saliva-derived samples, at least for the WGS applications. Each of these suggest that sequencing results obtained from saliva samples are comparable with those obtained from blood samples, at least in terms of sequencing accuracy. In addition, technical parameters of the sequencing data were also comparable, with certain anticipated differences, such as total read numbers (needed to achieve comparable numbers of human-mappable reads) or properly mapped reads (resulting from higher portions of non-human mappable reads) slightly worse in saliva samples. Contrary to Herzig et al. [13], we see shorter fragment lengths in saliva samples compared to blood samples. Inspecting the gDNA isolates (Additional File 2, Fig. 1), we saw partially fragmented gDNA in saliva samples. These fragments may have originated from gDNA isolation procedure. We used the same isolation kit for blood samples, where we have not seen these fragments. Hence, we suggest that these gDNA fragments are naturally present in saliva samples due to the aggressive microenvironment in the mouth, which naturally supports cell lysis and subsequent DNA degradation. During the NGS library preparation, which includes the fragmentation step, the naturally present gDNA fragments in saliva samples are further fragmented, resulting in shorter fragment lengths in saliva NGS libraries. Contrary to the effect of biological source, we proved a substantially higher effect of variant types and genome context on results accuracy. In general, SNVs resulted in higher accuracy than small-indels in the majority of analyzed genomic regions, which is a known phenomenon [52]. However, problematic genomic regions led to lower concordance rates for SNVs than for small-indels, but only in the WGS results (WGS_HARD). Restricting the genomic region of interest from WGS either to WGS_HCR or to WGS_NDR (or from WES to WES_HCR and WES_NDR) led to the most significant increase in median F1 scores. As could be anticipated, the lowest F1 scores were achieved in WGS_HARD and WES_HARD regions, both for SNVs as well as for small-indels. In line with this, plotting sequencing coverage, accuracy, and variant distributions against chromosomal locations revealed the non-uniform distribution of F1 scores throughout the genome. On the other hand, the patterns of fluctuations of F1 scores were highly similar between the RS NA12878 triplicates and the blood–saliva pairs. In both cases, reductions in F1 scores, fluctuations of sequencing depth, total numbers of identified variants, and unique variants for blood or saliva samples, tended to cluster with each other and with difficult-to-sequence regions of the genome. All these pattern similarities suggest that the decline in variant identification accuracy is not due to different properties (or different suitability) of the source biological material, but tends to cluster in difficult-to-sequence regions of the genome. Such clustering was also described previously; for example, on plasma-derived DNA samples [53]. Therefore, it is tied to the combined factors of the properties of these genomic sequences and the used sequencing technology itself, and it is difficult to determine whether the increase in identified variant numbers is due to real variants or artifacts. However, they are more numerous in regions where F1 score distributions indicate lower accuracy, even within the RS NA12878 triplicates. Since even the triplicates differ from each other in these genomic regions, these variants are rather artifacts generated during the sequencing process. In general, problematic genomic regions, representing challenges for sequencing and bioinformatic processing of sequenced data, should be further characterized in higher depths in dedicated study using, for example, different sequencing technologies.

All these results underline recent recommendations on the validation of sequencing protocol performance for different variant types as well as on different regions of the genome [29]. It should be taken into consideration, therefore, that the results may not apply to other variant types such as CNVs and TRs. On the other hand, TRs are represented in conventional variant calling as small-indels; therefore, the small-indel group may contain a variation of TR motifs that are associated with higher sequencing error rates [52]. Excluding TR motifs from conventional variant calling steps and using dedicated TR genotyping tools for these specific regions—such as HipSTR [54], Expansion Hunter [55], or Dante [26]—may hypothetically increase the accuracy of variant calling of small-indels. However, the viability of such an approach was not tested in this study. Its possibilities and limitations will be further studied in dedicated validation analyses.

When selecting biological material for NGS sequencing, one of the additional aspects to consider is the possible presence of contamination of human gDNA by other sources of nucleic acids. In the majority of cases, these are non-human sequences, mostly belonging to the microbiome, and in the case of saliva, mainly the human oral microbiome [56, 57]. This study proved that saliva-derived gDNA samples may contain a relatively large portion of sequences that do not map to the actual human reference genome (on average, 22% for WGS saliva samples) despite dedicated attention to unifying the sample collection procedure. Although these sequences may contain reads from the yet-missing parts of the human reference genome, relatively large portions of reads seem to belong to non-human DNA mapping; for example, to sequences mapping to the Human Oral Microbiome Database (HOMD). It is now widely discussed that blood samples may contain microbiome sequences even in healthy individuals, but these are possibly associated with certain diseases [58]. Furthermore, human oral microbiome analysis is very important in the evaluation of the overall health of the individual [59]. However, the blood samples in the study contained proportions of human-mappable reads and HOMD-mappable reads comparable with RS NA12878, raising questions such as whether these are yet-unmappable parts of the human genome, real human-oral-microbiome-based reads, or whether they arise from contamination sources such as kitomes [60–63]. However, in saliva, the proportion of non-human-mappable reads was much larger than in the blood or RS NA12878 and was highly variable, again much more than in the case of blood-derived gDNA. Fluctuations of F1 scores between samples were relatively low; however, one sample (sample_05) had lower F1 scores both in WGS and WES protocols, while it was also found to have the lowest human-mappable read numbers (i.e., the largest contamination rate) in the NovaSeq 6000 experiment. Nevertheless, no correlation was identified between contamination rate and lower sequencing accuracy when analyzing all of the samples. To compensate on reads lost because of the presence of non-human genomic material, and to achieve certain minimum coverage of human-mappable reads, and thus allow more efficient pooling of sequencing libraries, a low-pass sequencing step was incorporated during the library preparation, similar to Sosonkina et al. [64]. The use of such an additional step is the decision of the users, as it represents additional costs and time.

It should be noted, however, that our study, despite its complexity, has certain limitations. The majority of our conclusion based on the identified correlations, or lack of correlations, should be confirmed on a larger sample set, or even in slightly modified study designs. For the routine clinical usage of saliva samples, it would be interesting to extend the evaluation by including biological replicates from the same individuals at the same time, and also from the same individuals at different time points. Extending evaluations into other types of sequence variants, such as TRs, CNVs, or other structural variants, should also be performed, as well as a more detailed characterization of the effect of different genomic regions with respect to different variant types. Since they represent highly relevant parts of the genome, problematic genomic regions should also be characterized in more detail or even with different sequencing technologies. Since reliable benchmarking datasets are not generally available for these problematic genomic regions this could be challenging.

## Conclusion

Similarities in the sequencing accuracy and the distribution of different patterns of inaccuracies throughout the genome, together with results obtained when comparing other technical characteristics of the sequencing data, suggest that saliva-derived gDNA may be considered an equivalent material to blood-derived gDNA for WGS and WES analysis. When considering non-human-mappable reads, they were found to be present in relatively large proportions in the saliva-derived gDNA when compared with blood-derived gDNA, despite a strict sampling protocol. Although microbiome-to-human misalignment cannot be equivocally ruled out, the results suggest that the end effect does not deviate sequencing accuracy from values typically obtained using blood-derived gDNA. The decline in variant identification accuracy tends to cluster in the difficult-to-sequence regions of the genome, i.e., it is more likely tied to a combined effect of the properties of the genomic context, variant types, and the sequencing technology used. When evaluating the reliability of sequencing results, such as pathogenic variants identified, different quality control metrics should be considered. The study results suggest that although they are yet not generally used, information about the genomic region of the identified variant and the type of the sequencing protocol should be taken into account as well as other conventionally considered factors.

### Supplementary Information

**Additional file 1: Table 1. Table 2. Table 3. Table 4. Table 5. Table 6.**

**Additional file 2:** **Fig. 1.** Analysis of gDNA isolates integrity. The 0.8% agarose gel electrophoresis (GelRed 1:10 000, 100 V, 40 minutes). 1 kb; Ladder (c.n N3232L, New England Biolabs), Upper gel; DNA isolates 1-10 from blood 1-10, Lower gel; DNA isolates from saliva. 50 ng in 5 μl of each isolate were loaded/lane. The Gel was exposed to UV light and the picture taken with a gel documentation system Carestream Gel Logic 212 PRO and Carestream Molecular Imaging software (version 5.3.2.16673). The final picture was combined from 4 original pictures (The Additional File 2, Fig. 4) and postprocessed by adobe Photoshop version 8.0 using a tool: Cropping of the selected area, Brightness/Contrast (applied for a whole original picture), Horizontal and vertical type tool (to describe the samples and ladder). **Fig. 2.** Coverage, variant, and F1 score distributions throughout the human reference genome. Characteristics are clustered by 1,000,000 bases. Gray regions outline problematic genomic regions (HARD). The axis of each red track corresponds to low values to high values from the inside to the outside. The axis of each blue tract corresponds to low values to high values from the outside. Track numbering form the inner: Track 1: Average genome coverage of saliva (blue) and blood (red) samples. Reads filtered for quality>; Track 2: Average count of variants unique for saliva (blue) samples and variants unique for blood (red) samples; Track 3: Average count of all variants in saliva (blue) samples and all variants in blood (red) samples; Track 4: Median F1 score of comparisons between RS NA12878 repetitions; Track 5: Median F1 score of saliva versus blood comparisons. A) Whole genome view (excluding gonosomes); B) Zoom on chromosome 1. **Fig. 3.** Evaluation of WGS read mapping results with a focus on the mappability to the GRCh38 human reference genome (Human) and on the HOMD, for the final NovaSeq6000 sequencing. A) Saliva/Blood-derived gDNA WES on NovaSeq 6000 visualized as proportions of reads; B) Saliva/Blood-derived gDNA WGS on NovaSeq 6000 visualized as proportions of reads; HOMD = Human Oral Microbiome Database. **Fig. 4.** Analysis of gDNA isolates integrity (original pictures). The 0.8% agarose gel electrophoresis (GelRed 1:10 000, 100 V, 40 minutes). 1 kb; 1 kb Ladder (c.n N3232L, New England Biolabs), A; DNA isolates 2, ,3, 5, ,7, 8, 9 10 from blood. B; DNA isolates 1, 4 ,6 from blood. C; DNA isolates 2, 3 ,5 ,7 8, 9 from saliva. D; DNA isolates 1, 4, 6, 10 from saliva. 50 ng in 5 μl of each isolate were loaded/lane. The gels were exposed to UV light and the picture taken with a gel documentation system Carestream Gel Logic 212 PRO and Carestream Molecular Imaging software (version 5.3.2.16673). **Fig 5.** Analysis of **g**enotype concordance of the TDS and the three RS NA12878 iterations. A; results for SNVs in the WGS protocol (total number of variants 3 247 528), B; results for small-indels in the WGS protocol (total number of variants 483 332), C; results for SNVs in the WES protocol (total number of variants 25 298), D; results for small-indels in the WES protocol (total number of variants 891). All concordance rate calculations were limited to autosomes and HCR. Blue frames indicate variants found only in TDS and all three RS NA12878 iterations, respectively.

## Data Availability

In compliance with personal data protection, human genomic data from this study will be provided by the corresponding author upon a reasonable request, including the comparison of the study data with the third-party data and verification of the in silico protocol. Human genomic data will be shared upon request via a secure encrypted cloud link. All RS NA12878 raw sequencing data are available in ncbi bioproject PRJNA1008349 [65].

## Abbreviations

BED: Browser extensible data
CNV: Copy number variant
GIAB: Genome in a Bottle
HCR: High-confidence region
HOMD: Human Oral Microbiome Database
MAPQ10: Mapping quality 10
RS: Reference standard
S: Saliva
Indel: Insertion/deletion
NDR: Non-difficult regions
TDS: Truth dataset (ground truth variants in NA12878 reference sample)
TR: Tandem repeat
WB: Whole blood
WES: Whole exome sequencing
WGS: Whole genome sequencing
