# Supplementary figures and images for: Validated WGS and WES protocols proved saliva-derived gDNA as an equivalent to blood-derived gDNA for clinical and population genomic analyses
Source: BMC Genomics. 2024 Feb 17;25:187. doi: 10.1186/s12864-024-10080-0 (PMC10873937; doi:10.1186/s12864-024-10080-0)

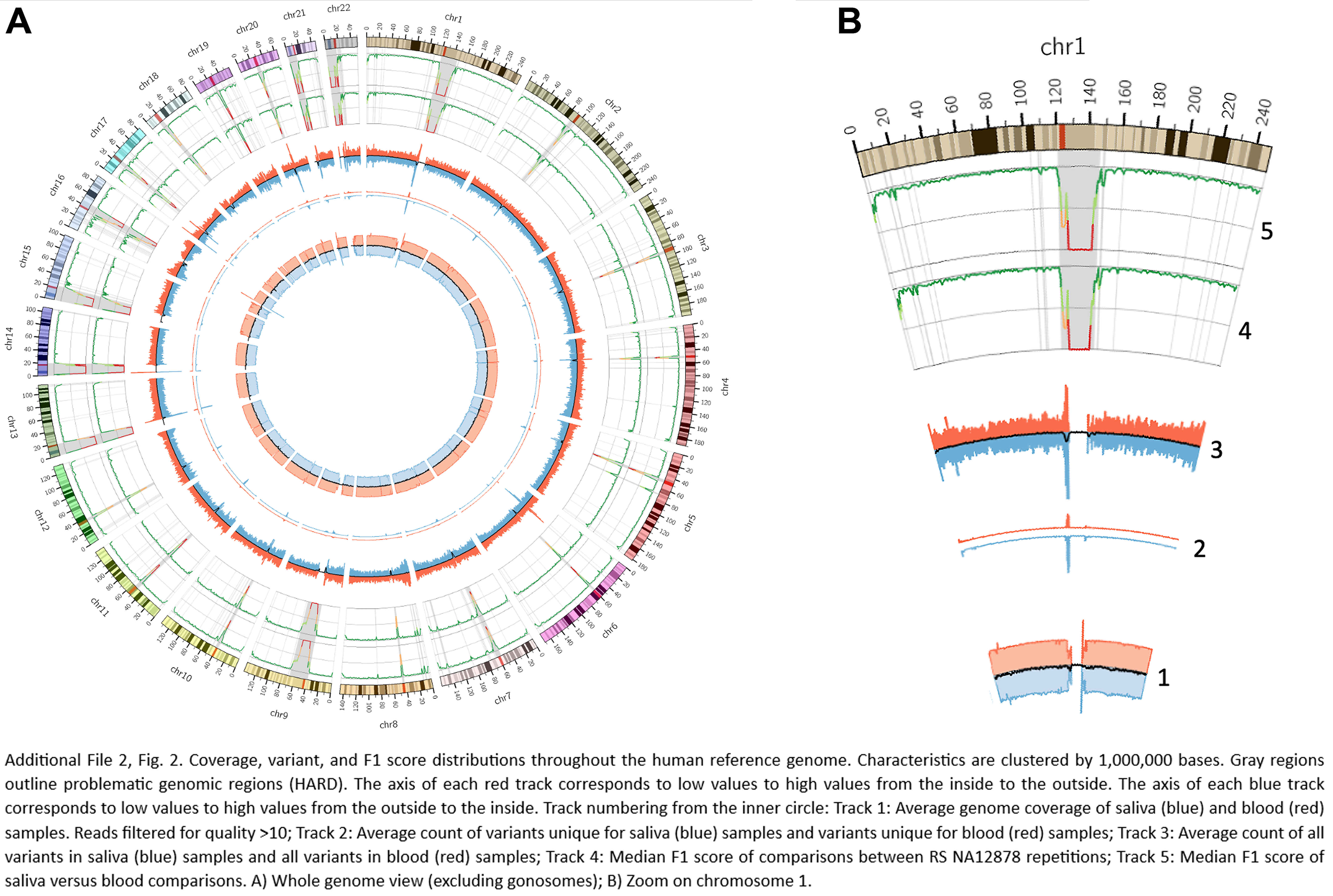

Supplement: Supplementary file 2 — Additional file 2: Fig. 1. Analysis of gDNA isolates integrity. The 0.8% agarose gel electrophoresis (GelRed 1:10 000, 100 V, 40 minutes). 1 kb; Ladder (c.n N3232L, New England Biolabs), Upper gel; DNA isolates 1-10 from blood 1-10, Lower gel; DNA isolates from saliva. 50 ng in 5 μl of each isolate were loaded/lane. The Gel was exposed to UV light and the picture taken with a gel documentation system Carestream Gel Logic 212 PRO and Carestream Molecular Imaging software (version 5.3.2.16673). The final picture was combined from 4 original pictures (The Additional File 2, Fig. 4) and postprocessed by adobe Photoshop version 8.0 using a tool: Cropping of the selected area, Brightness/Contrast (applied for a whole original picture), Horizontal and vertical type tool (to describe the samples and ladder). Fig. 2. Coverage, variant, and F1 score distributions throughout the human reference genome. Characteristics are clustered by 1,000,000 bases. Gray regions outline problematic genomic regions (HARD). The axis of each red track corresponds to low values to high values from the inside to the outside. The axis of each blue tract corresponds to low values to high values from the outside. Track numbering form the inner: Track 1: Average genome coverage of saliva (blue) and blood (red) samples. Reads filtered for quality>; Track 2: Average count of variants unique for saliva (blue) samples and variants unique for blood (red) samples; Track 3: Average count of all variants in saliva (blue) samples and all variants in blood (red) samples; Track 4: Median F1 score of comparisons between RS NA12878 repetitions; Track 5: Median F1 score of saliva versus blood comparisons. A) Whole genome view (excluding gonosomes); B) Zoom on chromosome 1. Fig. 3. Evaluation of WGS read mapping results with a focus on the mappability to the GRCh38 human reference genome (Human) and on the HOMD, for the final NovaSeq6000 sequencing. A) Saliva/Blood-derived gDNA WES on NovaSeq 6000 visualized as pro [file 12864_2024_10080_MOESM2_ESM.zip › additional file 2/Additional File 2, Fig 2.png]

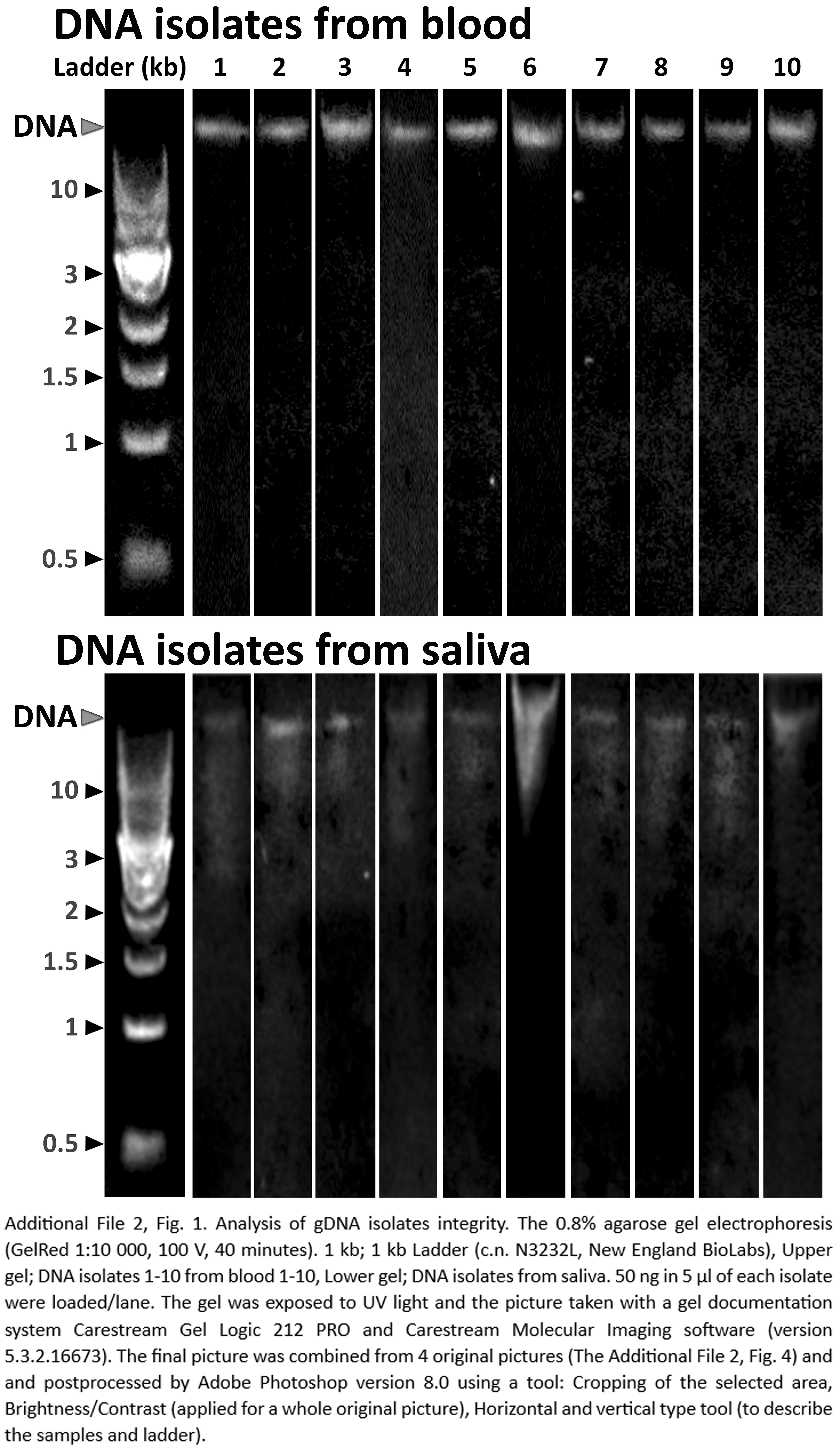

Supplement: Supplementary file 2 — Additional file 2: Fig. 1. Analysis of gDNA isolates integrity. The 0.8% agarose gel electrophoresis (GelRed 1:10 000, 100 V, 40 minutes). 1 kb; Ladder (c.n N3232L, New England Biolabs), Upper gel; DNA isolates 1-10 from blood 1-10, Lower gel; DNA isolates from saliva. 50 ng in 5 μl of each isolate were loaded/lane. The Gel was exposed to UV light and the picture taken with a gel documentation system Carestream Gel Logic 212 PRO and Carestream Molecular Imaging software (version 5.3.2.16673). The final picture was combined from 4 original pictures (The Additional File 2, Fig. 4) and postprocessed by adobe Photoshop version 8.0 using a tool: Cropping of the selected area, Brightness/Contrast (applied for a whole original picture), Horizontal and vertical type tool (to describe the samples and ladder). Fig. 2. Coverage, variant, and F1 score distributions throughout the human reference genome. Characteristics are clustered by 1,000,000 bases. Gray regions outline problematic genomic regions (HARD). The axis of each red track corresponds to low values to high values from the inside to the outside. The axis of each blue tract corresponds to low values to high values from the outside. Track numbering form the inner: Track 1: Average genome coverage of saliva (blue) and blood (red) samples. Reads filtered for quality>; Track 2: Average count of variants unique for saliva (blue) samples and variants unique for blood (red) samples; Track 3: Average count of all variants in saliva (blue) samples and all variants in blood (red) samples; Track 4: Median F1 score of comparisons between RS NA12878 repetitions; Track 5: Median F1 score of saliva versus blood comparisons. A) Whole genome view (excluding gonosomes); B) Zoom on chromosome 1. Fig. 3. Evaluation of WGS read mapping results with a focus on the mappability to the GRCh38 human reference genome (Human) and on the HOMD, for the final NovaSeq6000 sequencing. A) Saliva/Blood-derived gDNA WES on NovaSeq 6000 visualized as pro [file 12864_2024_10080_MOESM2_ESM.zip › additional file 2/Additional file 2, Fig. 1.png]

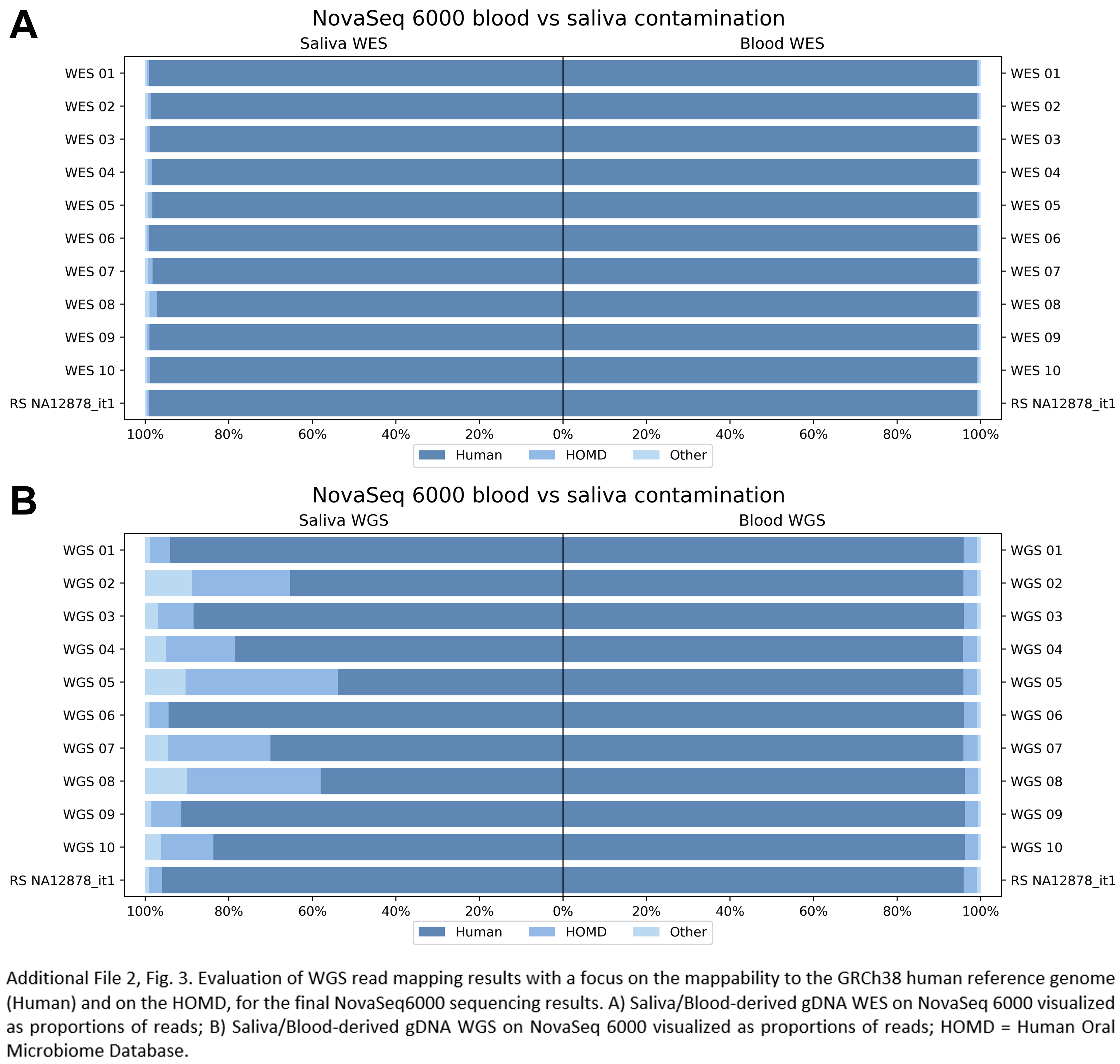

Supplement: Supplementary file 2 — Additional file 2: Fig. 1. Analysis of gDNA isolates integrity. The 0.8% agarose gel electrophoresis (GelRed 1:10 000, 100 V, 40 minutes). 1 kb; Ladder (c.n N3232L, New England Biolabs), Upper gel; DNA isolates 1-10 from blood 1-10, Lower gel; DNA isolates from saliva. 50 ng in 5 μl of each isolate were loaded/lane. The Gel was exposed to UV light and the picture taken with a gel documentation system Carestream Gel Logic 212 PRO and Carestream Molecular Imaging software (version 5.3.2.16673). The final picture was combined from 4 original pictures (The Additional File 2, Fig. 4) and postprocessed by adobe Photoshop version 8.0 using a tool: Cropping of the selected area, Brightness/Contrast (applied for a whole original picture), Horizontal and vertical type tool (to describe the samples and ladder). Fig. 2. Coverage, variant, and F1 score distributions throughout the human reference genome. Characteristics are clustered by 1,000,000 bases. Gray regions outline problematic genomic regions (HARD). The axis of each red track corresponds to low values to high values from the inside to the outside. The axis of each blue tract corresponds to low values to high values from the outside. Track numbering form the inner: Track 1: Average genome coverage of saliva (blue) and blood (red) samples. Reads filtered for quality>; Track 2: Average count of variants unique for saliva (blue) samples and variants unique for blood (red) samples; Track 3: Average count of all variants in saliva (blue) samples and all variants in blood (red) samples; Track 4: Median F1 score of comparisons between RS NA12878 repetitions; Track 5: Median F1 score of saliva versus blood comparisons. A) Whole genome view (excluding gonosomes); B) Zoom on chromosome 1. Fig. 3. Evaluation of WGS read mapping results with a focus on the mappability to the GRCh38 human reference genome (Human) and on the HOMD, for the final NovaSeq6000 sequencing. A) Saliva/Blood-derived gDNA WES on NovaSeq 6000 visualized as pro [file 12864_2024_10080_MOESM2_ESM.zip › additional file 2/Additional File 2, Fig. 3.png]

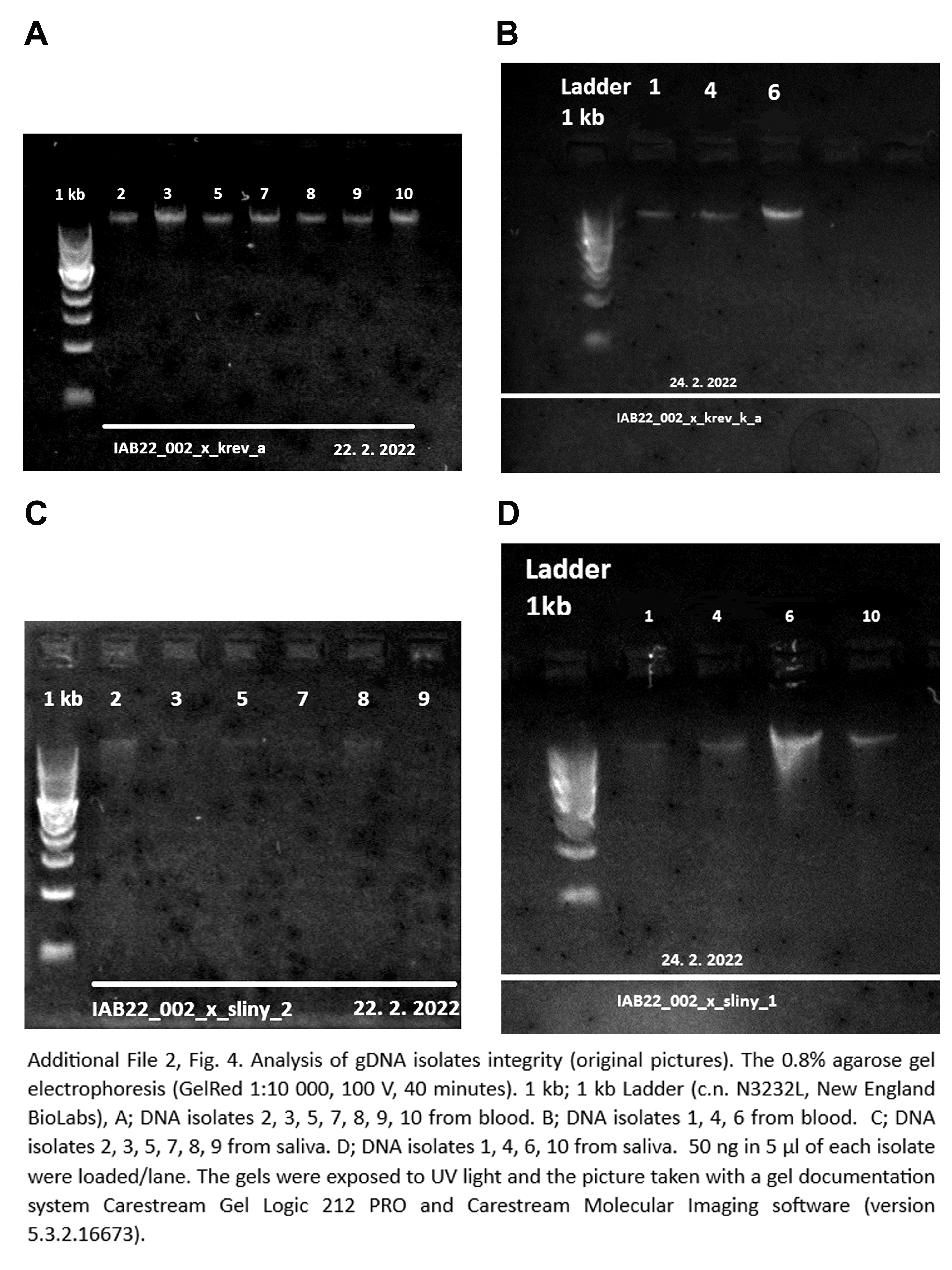

Supplement: Supplementary file 2 — Additional file 2: Fig. 1. Analysis of gDNA isolates integrity. The 0.8% agarose gel electrophoresis (GelRed 1:10 000, 100 V, 40 minutes). 1 kb; Ladder (c.n N3232L, New England Biolabs), Upper gel; DNA isolates 1-10 from blood 1-10, Lower gel; DNA isolates from saliva. 50 ng in 5 μl of each isolate were loaded/lane. The Gel was exposed to UV light and the picture taken with a gel documentation system Carestream Gel Logic 212 PRO and Carestream Molecular Imaging software (version 5.3.2.16673). The final picture was combined from 4 original pictures (The Additional File 2, Fig. 4) and postprocessed by adobe Photoshop version 8.0 using a tool: Cropping of the selected area, Brightness/Contrast (applied for a whole original picture), Horizontal and vertical type tool (to describe the samples and ladder). Fig. 2. Coverage, variant, and F1 score distributions throughout the human reference genome. Characteristics are clustered by 1,000,000 bases. Gray regions outline problematic genomic regions (HARD). The axis of each red track corresponds to low values to high values from the inside to the outside. The axis of each blue tract corresponds to low values to high values from the outside. Track numbering form the inner: Track 1: Average genome coverage of saliva (blue) and blood (red) samples. Reads filtered for quality>; Track 2: Average count of variants unique for saliva (blue) samples and variants unique for blood (red) samples; Track 3: Average count of all variants in saliva (blue) samples and all variants in blood (red) samples; Track 4: Median F1 score of comparisons between RS NA12878 repetitions; Track 5: Median F1 score of saliva versus blood comparisons. A) Whole genome view (excluding gonosomes); B) Zoom on chromosome 1. Fig. 3. Evaluation of WGS read mapping results with a focus on the mappability to the GRCh38 human reference genome (Human) and on the HOMD, for the final NovaSeq6000 sequencing. A) Saliva/Blood-derived gDNA WES on NovaSeq 6000 visualized as pro [file 12864_2024_10080_MOESM2_ESM.zip › additional file 2/Additional file 2, Fig. 4.png]

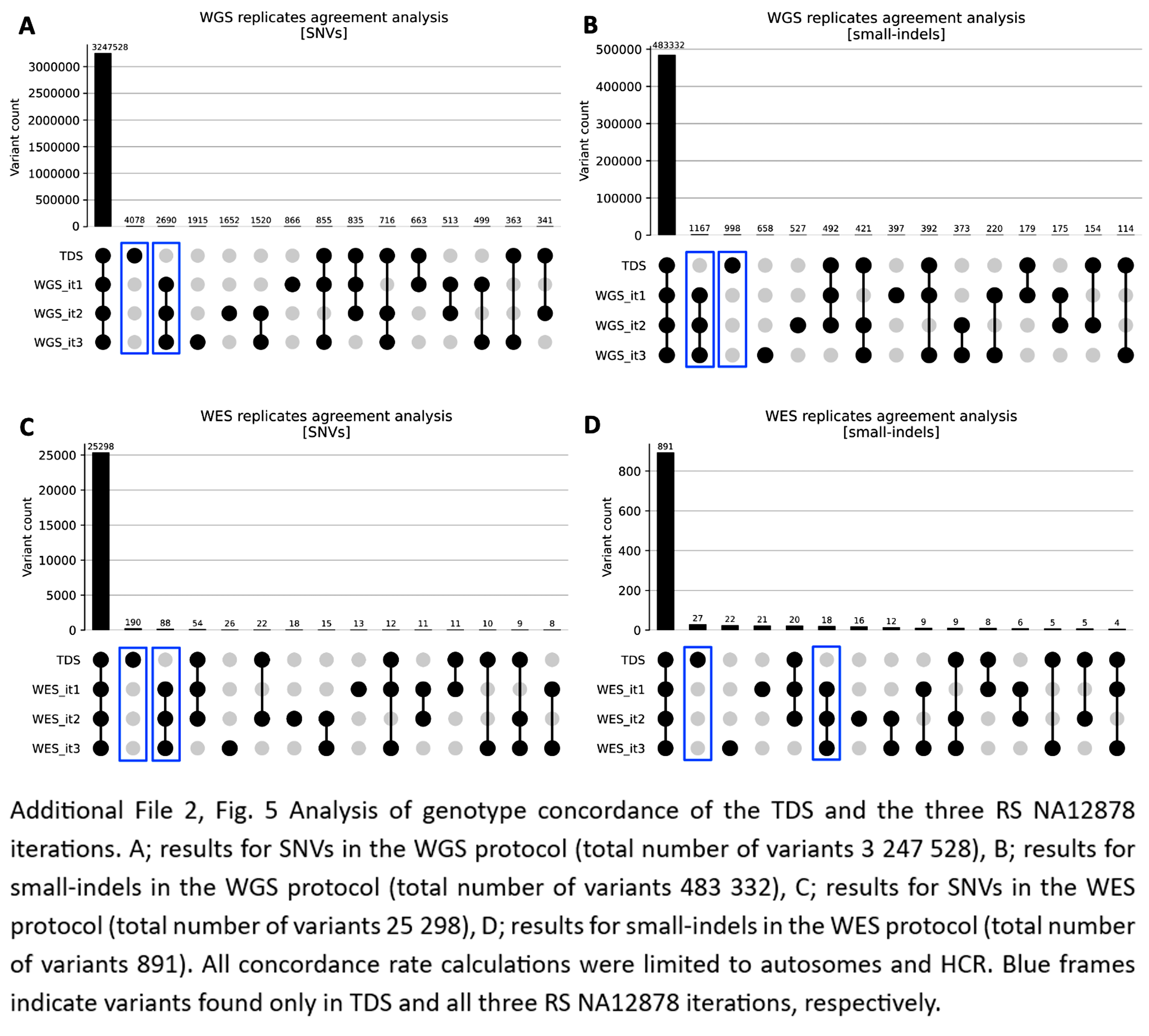

Supplement: Supplementary file 2 — Additional file 2: Fig. 1. Analysis of gDNA isolates integrity. The 0.8% agarose gel electrophoresis (GelRed 1:10 000, 100 V, 40 minutes). 1 kb; Ladder (c.n N3232L, New England Biolabs), Upper gel; DNA isolates 1-10 from blood 1-10, Lower gel; DNA isolates from saliva. 50 ng in 5 μl of each isolate were loaded/lane. The Gel was exposed to UV light and the picture taken with a gel documentation system Carestream Gel Logic 212 PRO and Carestream Molecular Imaging software (version 5.3.2.16673). The final picture was combined from 4 original pictures (The Additional File 2, Fig. 4) and postprocessed by adobe Photoshop version 8.0 using a tool: Cropping of the selected area, Brightness/Contrast (applied for a whole original picture), Horizontal and vertical type tool (to describe the samples and ladder). Fig. 2. Coverage, variant, and F1 score distributions throughout the human reference genome. Characteristics are clustered by 1,000,000 bases. Gray regions outline problematic genomic regions (HARD). The axis of each red track corresponds to low values to high values from the inside to the outside. The axis of each blue tract corresponds to low values to high values from the outside. Track numbering form the inner: Track 1: Average genome coverage of saliva (blue) and blood (red) samples. Reads filtered for quality>; Track 2: Average count of variants unique for saliva (blue) samples and variants unique for blood (red) samples; Track 3: Average count of all variants in saliva (blue) samples and all variants in blood (red) samples; Track 4: Median F1 score of comparisons between RS NA12878 repetitions; Track 5: Median F1 score of saliva versus blood comparisons. A) Whole genome view (excluding gonosomes); B) Zoom on chromosome 1. Fig. 3. Evaluation of WGS read mapping results with a focus on the mappability to the GRCh38 human reference genome (Human) and on the HOMD, for the final NovaSeq6000 sequencing. A) Saliva/Blood-derived gDNA WES on NovaSeq 6000 visualized as pro [file 12864_2024_10080_MOESM2_ESM.zip › additional file 2/Additional File 2, Fig. 5.png]
